# Supplementary material for: Assessing the impact of introductory programming workshops on the computational reproducibility of biomedical workflows
Source: PLoS One. 2020 Jul 8;15(7):e0230697. doi: 10.1371/journal.pone.0230697 (PMC7343163; doi:10.1371/journal.pone.0230697)
Supplement: S1 Checklist — (PDF) [file pone.0230697.s001.pdf]

Participant ID: ___________ Ariel Deardorff, August 12, 2019

Reproducibility Score Card

Note: Researchers are given 1pt for each yes to calculate a total score

As part of your research workflow do you currently or intend to:

Question	Pre-Workshop (Y/N)	Post-Workshop (Y/N)
Use programming languages like R, Python, or the command line for data acquisition, processing, or analysis?		
Transform step-by-step workflows into scripts or functions?		
Use version control[1] to manage code?		
Use open source software?		
Share your code publicly[2]?		
Share your computational workflow or protocols publicly?		
**Total Score**	/6	/6

[1] Manual version control doesn't count
[2] Publicly means "with a wider research audience outside UCSF"
