# Supplementary material for: Assessing the impact of introductory programming workshops on the computational reproducibility of biomedical workflows
Source: PLoS One. 2020 Jul 8;15(7):e0230697. doi: 10.1371/journal.pone.0230697 (PMC7343163; doi:10.1371/journal.pone.0230697)
Supplement: S1 File — (PDF) [file pone.0230697.s002.pdf]

Participant ID: _____________

Ariel Deardorff, August 12, 2019

Data Acquisition/Input

Data Processing

Data Analysis

Research Outputs
