# Supplementary material for: Assessing the impact of introductory programming workshops on the computational reproducibility of biomedical workflows
Source: PLoS One. 2020 Jul 8;15(7):e0230697. doi: 10.1371/journal.pone.0230697 (PMC7343163; doi:10.1371/journal.pone.0230697)
Supplement: S2 File — (PDF) [file pone.0230697.s003.pdf]

Interview Protocols

Ariel Deardorff
January 14, 2019

Pre-Workshop Interview

Items to bring to the interview:

- This interview protocol!
- Notepad for misc. notes
- Multiple pens – some red
- Example workflow templates
- Workflow templates + blank papers
- Reproducibility scorecard
- Recording Equipment
- 2 Consent forms (one for the participant to keep)
- Giftcard

Remember to refresh memory on their name, status, area of research and the workshop they registered for!

Intro

My name is Ariel Deardorff and I am a Data Librarian here at the UCSF Library. My work is focused on helping researchers make their work more efficient and reproducible – especially when it comes to analyzing and visualizing their data.

The goal of this study is to examine the impact of the Library's introductory programming workshops on your research process. In particular, we want to see if the skills taught in the workshops enable you to incorporate more computational methods into your workflow.

Today we are going to spending most of our time drawing and discussing your current research process to learn more about the tools and methods that you use right now.

During our second interview in June we will take another look at your research process and examine any changes to see whether our programming workshops had an impact on your research workflow.

Consent Form

Before we get started I want to make sure you understand that this conversation will be recorded. Your personal data will be kept confidential although anonymized transcripts and data may be shared alongside research publications. You are free to skip questions and we can stop at any time. If that all sounds good can I get you to sign this form?

Turn on recorder!

Before we dive into the workflow, can you tell me a little bit about your area of research? What do you study and what are the goals you are hoping to achieve with your work? (*Limit this to 5 minutes)*

Workflow Diagramming

[Current research process – 40 min]

Now let's talk about your current research process in more detail by drawing your research workflow.

A research workflow is all the steps and processes you go through from data collection to producing the end product, whether that is a paper, poster, or code.

I have divided this paper into roughly four sections:

- Data Acquisition: data input or creation, involving gathering data in raw format, either via instruments, or acquiring data from existing sources: ends in raw data
- Data Processing: cleaning data so that it is fully usable for data analysis (might include QA checks, excluding nulls, etc)
- Data Analysis: data visualization, statistical analysis, creating figures for publication
- Research Outputs: the products you produce from your research that you share with others

Here are some example workflows to give you an idea what these might look like. Keep in mind that yours might look very different and you might not be involved with every stage of the research process. *Show two examples from* https://www.practicereproducibleresearch.org/

Now I would like you to draw your workflow, please talk aloud as you draw, and we can discuss the tools, methods, and processes that you use. The idea here is to get your workflow down on paper so we can compare it to your workflow 3 months after the workshop and note changes. We will probably spend about 30-40 minutes drawing and discussing the diagram.

As they draw prompt them to discuss:

- A description of each stage – what they do and why
- The tools they use (are they open source?)
- If they are using code at any point,
    - Who wrote the code?
    - What is the task? (Are they using it to turn step-by-step workflows into a pipeline?)
    - What part of the data lifecycle? (acquisition, processing, analysis)
    - Is it version controlled?
    - Do they plan to share any of the code? Where?
- Protocol/workflow sharing

If they mention pain points/wanting to improve something – get into it!

- What about that is frustrating?
- Is that something they are hoping to address in SWC?

After they have described each stage in the process, prompt them with:

- Do you feel like you have covered every step in your research workflow?
- Are there any other tools that you use in the context of your research that aren't recorded here?

[Pain points – 20 min]

Now I want to finish up by talking about your thoughts on your current workflow.

- Is there any part of this workflow that feels frustrating or not ideal to you?
    - What are they? (mark in red) *Make sure they describe aloud*
        - What about that is frustrating?
        - Is that something you are hoping to address in SWC?
- What are you hoping to learn in SWC?
- Is there anything else you would like to share about your research workflow?

Checklist time!

Now I have a couple quick yes/no questions to summarize your workflow. These are behaviors that software carpentry (the organization we work with to teach our programming workshops) hopes to teach you, and we want to get a sense of what you are doing now. As part of your research workflow do you currently or have a plan to:

[checklist]

Thank you so much for speaking with me today about your research! I learned a lot! I look forward to meeting with you again in June after the workshop to see how things have changed.

Give Giftcard!

Afterwards:

- Upload the audio file from the recorder
- Photograph and upload the diagram, consent form, and scorecard
