# Supplementary material for: Assessing the impact of introductory programming workshops on the computational reproducibility of biomedical workflows
Source: PLoS One. 2020 Jul 8;15(7):e0230697. doi: 10.1371/journal.pone.0230697 (PMC7343163; doi:10.1371/journal.pone.0230697)
Supplement: S3 File — (PDF) [file pone.0230697.s004.pdf]

Post Workshop Interview Protocol

Ariel Deardorff
June 4, 2019

Items to bring to the interview:

- This interview protocol!
- Notepad for misc. notes
- Multiple pens – pink & green highlighter
- Their original workflow + blank papers
- Their reproducibility scorecard
- Interview summary page
- Recording Equipment
- Giftcard

Turn on recorder!

Reflections on the Workshop

[to refresh their memory on the workshop and learn if there are ways we can improve it for future learners, also start to get at barriers/enablers of learning to program]

Let’s start by talking about what you thought of the programming workshop in March

- Were you able to attend both days?
- What did you think of the workshop?
- What was your biggest takeaway?
- What would have made it a better learning experience for you?

Change in their Research Workflow

[to learn about the changes they have made and plan to make to their workflows (and the impact of these changes), and what factors enabled or prevented their success]

Summarize Changes

Now let's take a look at your drawing of your research workflow. *Look at it together and summarize it together (including pain points)*

Now that it has been 3 months since the workshop, have you made any changes to your workflow? *Participants mark changes on their original diagram in pink highlighter, as they do discuss:*

- **What was the change?**
  - *New tool? Method?*
  - *In what part of the workflow?*
  - *Was this one of their original goals in learning to program?*
- **Why did you make that change?**
  - *What prompted/enabled it?*
  - *Dig into this!*
  - *Was it because of something they learned in SWC? – very important!*
- **How has that change impacted your research?**
  - *Are they able to do things better, faster, etc?*
  - *How does this make them feel?*

Future Plans – *whether or not they made changes, mark plans in green highlighter*

Do you have any ideas/plans for **future changes**? Is there anything you will do differently in future workflows?

- How do you think these changes will impact your research?

Focus on Pain Points – i*f not addressed earlier*

When we talked before the workshop you also mentioned these pain points *[discuss one at a time]*. Has anything changed related to those?

Focus on Goals – *if not addressed earlier*

When we chatted before you mentioned that you wanted to learn programming to do *[list and discuss one at a time].* Do you think you have made any progress on those goals?

Workflow wrap up

- Have you incorporated Unix, Git, or Python/R anywhere else in your workflow?
- Are there any other areas where you plan on making changes?
- Anything else you would like to share that is different about your research practices now?

Programming Support – Overall view

- What helped you integrate programming into your workflow?
  - *Try to get at all possible success enablers (supportive PI, relevant project, etc.)*
- What barriers did you encounter in implementing your new skills?

Reproducibility Checklist

[to compare pre and post behavior, get their thoughts on reproducibility]

To summarize your research practices let's run through that behavior checklist again one more time:

[checklist]

Follow up on anything new from the checklist that we didn't discuss

Reproducibility Question

Finally, one of our goals in teaching people to program is to help make their research more computationally reproducible, meaning that someone else could take your data and analysis code and produce the same results. Do you think participating in this workshop has made your work more reproducible? *Why/why not?*

Thank you so much for taking the time to speak with me about your work!
