# Supplementary material for: Assessing the impact of introductory programming workshops on the computational reproducibility of biomedical workflows
Source: PLoS One. 2020 Jul 8;15(7):e0230697. doi: 10.1371/journal.pone.0230697 (PMC7343163; doi:10.1371/journal.pone.0230697)
Supplement: Attachment — Submitted filename: ResponseToReviewers.docx [file pone.0230697.s006.docx]

**Response to Reviewers**

Ariel Deardorff

May 18, 2020

PONE-D-20-06264: Assessing the impact of introductory programming workshops on the computational reproducibility of biomedical workflows.

**Reviewer #1**

| **Reviewer Comments** | **Author Response** |
| --- | --- |
| A main concern is about the overall impact/power of this approach, especially keeping the following two points in mind: - The survey was performed on learners that participated in Software Carpentry workshops (although this point is not specifically mentioned in the text, I believe that this is an implied statement, also supported by the description of the workshops - page 6, line 125). - The Carpentries maintain a fairly rigorous impact assessment programme, with a number of reports and analyses available on their website ([https://carpentries.org/assessment/](https://urldefense.proofpoint.com/v2/url?u=https-3A__carpentries.org_assessment_&d=DwQGaQ&c=iORugZls2LlYyCAZRB3XLg&r=2w2LqNklovOgAfLOlchmAehWfoSIH-o4w5XbGtzEfPE&m=1rxF0Dtg3DrnFcNtmfpUStQETINgJowmZgUE_baS7KE&s=7gLxI3p7xbfLHFGq7bERV2mVD6jt08Tu-bhcsqxFNFc&e=)) under Zenodo DOIs, and including an "Analysis of Software and Data Carpentry’s Pre- and Post-Workshop Surveys" and a fairly recent "Analysis of The Carpentries Long-Term Surveys" (April 2020)  Although the work present here is much more detailed and focused on the reproducibility aspects (as opposed to the more streamlined and "templated" analysis that the Carpentries offer), at the same time the main driving point do share a significant overlap. Given that, due to the small sample size, the quantitative analysis lacks appropriate power, as also highlighted within the manuscript, it may be worth investigating how the interview data could be complemented by data gathered through the Carpentries impact assessment programme. In particular, this would hopefully underline the insights gained through this work, and connect to a larger number of research communities that are transitioning to more computational approaches (such as the Social Sciences, similarly to Life Sciences) | Thank you for your comments. These were indeed Software Carpentry workshops and I have added a note in the beginning to make that more clear. I have also updated the introduction to refer to the 2020 Carpentries Long-Term survey results (rather than the 2018 ones that were published at the time of my submission). Finally, I agree that it would add more weight to my analysis to add in data from the Carpentries long-term survey and I have added an additional paragraph to the Discussion section which compares the key themes of my interviews with selected results from the survey. |
| 2. Another point is with regards to the existing bias due to the academic background of the learners. Although there is a representation of several different levels within the cohort, there is a significant bias in the "postdoctoral researcher" level with significantly less participation from other levels. Although this is not surprising nor unexpected, it would also make sense to include a stratified analysis of the results as it would be interesting to see the differences in impact from the workshop at different levels of academia. | Given the small sample size I don’t think that a stratified analysis of the results is appropriate as there would be too few people per category (ex: just one graduate student, and one faculty member). That said, I think you are correct about bias towards postdocs and I have added an extra sentence to the limitations section reflecting this. |
| 3. A final point to highlight is that the actual analysis of the data (i.e. the computational notebook and/or the script) is not available. It is understood that the Dedoose platform was used for analyzing such data; however, I believe that, if the specific computational steps were available, it would greatly increase the further re-use of both data and insights gained through this work. | As the majority of this project was a qualitative data analysis I didn’t produce any scripts or a computational notebook. Thematic analysis consists of reading through and iteratively applying codes (meaning key terms rather than computer code) to transcripts. In order to make this process more transparent though, I have now uploaded versions of the transcripts with applied codes to Dryad (in addition to the codebook that was previously uploaded). Hopefully this will give more insight to the analysis process. |

**Reviewer #2**

| **Reviewer Comments** | **Author Response** |
| --- | --- |
| This is an interesting study. We all know that it is good for biomedical researchers to have some computing knowledge in order to make their research reproducible and make the right choice in terms of tools (and libraries), but I have not come across many studies that actually talk about it. Initially I was a bit worried about the sample and it's small size, in terms of statistical power, but then the authors have actually mentioned about them as limitations. So, readers will be aware of this when they read this. I checked all the forms etc uploaded in DRYAD. | Thank you for your comments and for verifying that the data was appropriately shared in Dryad. |
